# Supplementary material for: Beyond early initiation: A qualitative study on the challenges of hospital-based postpartum breastfeeding support
Source: PLOS Glob Public Health. 2022 Nov 8;2(11):e0001266. doi: 10.1371/journal.pgph.0001266 (PMC10021460; doi:10.1371/journal.pgph.0001266)
Supplement: S2 Table — (PDF) [file pgph.0001266.s002.pdf]

Additional file 4: Summary of qualitative results

Category	Code	Frequency of code	Illustrative excerpts
Breastfeeding initiation support	Counselling for initiation	21	"Ok, when she (mother) came with the baby…the nurse helped her and put the baby in her arms. Then she (the nurse) said, "Now, the baby must be hungry. You need to feed her." Then she talked for a long time and said, "Are you ready to start feeding the baby?" And then my sister said, "Yes" and she started feeding the baby." Aunt from a district hospital<br><br>"So I will say, my name is so, so, so, I am here to help you. Your baby needs breast milk for nutrition and then for you to feed your baby well you have to hold your baby like this. Ok, make sure the baby's head is above your elbow and your hand is below the baby's buttocks and make sure your other hand is holding the breast with four fingers down and the thumb on top of the nipple on top of the areola, the areola is the right part of your breast so you will be holding it with your thumb as gently to express the milk to your baby, make sure your baby is in that position throughout the breast feeding period as you are feeding the baby. So I think in that way I can help the mother to produce good results." Clinical officer from a district hospital<br><br>"This lactation support, I first found it being done in the post natal when I was just coming, because it is done soon after the birth of the baby and we have to support these mothers, how to breastfeed the baby, how to attach the baby and also tell them the importance of breastfeeding" Nursing officer from the tertiary hospital
	Delays in initiation	6	"There were some delays… for one day… It was because breast milk was not coming out." Mother from tertiary hospital<br><br>"Most of the times we try as much as possible to facilitate this woman to start breast feeding at the same time, but the challenge is most of the times milk may not be produced so the woman may be complaining that the milk is not being produced, what am I supposed to do, so we try this we try this, you find out two

			days has gone the woman is not breast feeding, so we tried many things, we found the it is not working." Clinical officer from a district hospital
	Descriptions of early initiation	34	"It did not take long after the baby was born… Just after the baby was born, it only took approximately fifteen minutes then they told me that the baby is supposed to exclusively breastfeed because when doing so, the uterus goes back to its position and it also helps the baby to be healthy." Mother from a district hospital<br><br>"I don't think it took long because when I came back with the porridge the baby was feeding already." Aunt from a district hospital<br><br>"Usually when women are coming out from labor and we have seen that they are just fine…they start breastfeeding right away… 30 minutes after delivery, the baby must be found being breastfed. The reason is [that] the first milk that comes out is very important because it contains colostrum and it has some antibodies so that that the baby should not fall sick frequently." Tertiary hospital nurse<br><br>"It should be started soon after the baby is born…. As soon as the baby is born, the baby should be put on the chest of the mother. The mother is fine is in stable condition the vital signs are ok, should start breast feeding immediately." Clinical officer from a district hospital
Post-initiation breastfeeding support	Barriers to post-initiation support	92	"They said we should breastfeed exclusively… [and] we should take care of the baby…that's all that l remember" Mother from a district hospital<br><br>"I couldn't call it refusing… You provide the information, they practice and everything, and they forget… I have never had an example of refusing someone who does not want to do this. But sometimes after intensive teaching, after intensive support, then you go there you find them back to the old ways of doing things they are used to." District hospital nurse<br><br>"What is normally focused on is: how is the mother? The mother is not bleeding; the mother is fine. How is the baby? The baby is fine [and] is breastfeeding well.

			Fine, the mother can go to the postnatal ward…uhh… We have left the mother who is not bleeding, who is fine, the vitals are fine and is breastfeeding and you just take it for granted that everything is alright but we don't know this. Is it (breastfeeding) really happening? Is the support the right one? Is it being done the right way? Something like that… mostly the mothers were just being left like that…So people didn't even bother even to go there (to postnatal) to do monitoring...” District hospital nurse<br><br>“The moment we do the health talk, we assume that every mother has understood. Unless she comes to ask for… individual assistance. We never go there to say, let me see how you are feeding the child, is your child feeding?” Tertiary hospital nurse-midwife<br><br>“From my observation, it is not monitored except for the two hours we had we have with the mother in labor, because after the mother delivers, you have to observe the mother for the first two hours, how she is faring and [then] you refer them to the recovery room (postnatal ward). So I would say the only time that we do the observations are these two hours… if you are in short [staffed] the moment, you finish with the mother, you give her the baby to breastfeed… you teach her, you leave her…you are moving that mother to the recovery so to me, I would say monitoring is not done…” Tertiary hospital nurse-midwife<br><br>“We had a baby who had birth asphyxia but the baby was pink and normal but it was still asphyxia. I think at some point, the baby had some twitching. You know, when we expressed the milk on the mother and the baby breastfed and after three meals we saw the baby changing (improving)… That time, we had only the nursery ward and we were not monitoring glucose level of these babies so we were just managing symptoms” District health officer<br><br>“We check them maybe after they have just delivered and started breastfeeding, in post natal ward would ask them to position the babies on the breast so that we monitor how the babies are being breastfed but when they are in the ward the monitoring isn't done. Monitoring might be done but is done but it's not like on a schedule so mostly the nurses just ask if the milk is being produced and if the

			baby is being breastfed but once they see that everything is normal they don't monitor again" District health officer<br><br>"The teen did not remind the nurse that she did not breast feed again. Being a new and young mom, we needed to observe her while breast feeding…[but] she did not remind the nurse. Unfortunately, there were only two nurses that day and they were so busy" Nurse from a district hospital<br><br>"It's a challenge because we are understaffed… so most of the time it is a problem to monitor lactation unless if we are assessing those with challenges…. Ideally, soon after mothers are admitted in the ward, they are supposed to be monitored on how they are holding the babies to the breast and if they are producing milk, and on consecutive assessments, we have to assess if they are producing milk and if they are holding the baby correctly to the breast… During the day, we have six nurses and three during the night and they are two postnatal wards, 120 beds in total… so this poses as a challenge to assess how the women are breastfeeding their babies" Tertiary hospital nursing officer<br><br>"Most of the time, we fail to monitor effectively because of a shortage of staff. We are very few which makes us just to monitor those cases that we believe are critical… For example, it happened to me, on that day, I had a patient who was bleeding and the other patient had convulsions. The situation made me be in dilemma and choose to leave women who were breastfeeding and attend those cases." Tertiary hospital nurse<br><br>"The health workers feel they are busy people…If I have helped a mother deliver, we are done and we move to another patient, and this makes us not to focus on following the mother…." District medical officer<br><br>"You try to give support to help the mother and everything, then you leave [and] the guardian, especially the elderly, come in [and] get the baby… The babies usually don't stay with their mothers. We push them for the babies [to stay] with their mothers [but] then the guardians think they love the patient (the baby) very much and they get the baby again for a long time. You try to push them to say,

			"No, no, this baby has to be staying with the mother almost all the time to help them when you leave." They say, "No, our patient is so sick". …yes, something like that, the baby is usually in the hands of guardians." District hospital nurse
	Enablers of post-initiation support	43	"When they (nurses) come they want to see you doing what you were taught; namely putting those fingers at right position and make the baby breast feed accordingly." Mother at a district hospital<br><br>"They explained that I should also make sure that baby is feeding because sometimes the mother gets tired and falls asleep" Grandmother at a district hospital<br><br>"They told us that the babies should be exclusively breastfed… Since she (the mother) is not well, they (health workers) are explaining everything to me…They told us that the babies should be in a good position when breastfeeding." Grandmother at a district hospital<br><br>"We encourage the guardian (mother's relative), if the mother is unable to breast feed the baby because of other condition, we encourage the guardian to express milk in a well clean cup, so that she can feed the baby" Clinical officer at a district hospital<br><br>"Most of the times, [the] caregivers here are the grandmothers who usually help the patient by taking the breast and feed the baby. They teach such mothers how to breastfeed. They also encourage the woman to keep on breastfeeding." Tertiary hospital nurse<br><br>"Including their guardians (mother's relative) when providing the support because they support them when you are not around" Nurse from a district hospital<br><br>"The idea is not that the nurse should be there all the time, so you train the mother. You empower the mother. You teach them and observe if they are following the norms…" Tertiary hospital consultant

	Descriptions of any harms	7	"Once we were on a night shift…she was a prim (first-time mother) and postnatal…. This one was breastfeeding the baby while she is asleep…so [she] was told that that is bad and that is not the way to do it, because the baby may choke on the milk…. So, when we came on night shift...we also reminded her that "No no, when you are breastfeeding the child, make sure you are sitting. If you sit, then you breastfeed the child [and]…you may sleep". So, it happened that, it was around AM hours, the guardian (female relative) knocked and said come and see the baby. When we went there, we found the baby. I think the baby had aspirated. So, what was coming out was milk mixed with blood from the nose and the mouth. Then, we rushed the baby to the nursery. When we got there, they tried resuscitation but it didn't do us any good. That's how the baby died. Now, the guardian started accusing the child (the mother) … So, when this was happening, the mother felt it was normal. When the child was like coughing, coughing, she thought it was normal, but that time, I think that's when the baby had aspirated…. The baby was trying to tell her that "I am not fine." Yea, that's how it ended so it was sad and then it was like everybody in the morning was accusing her (the mother), that she didn't follow what she was told, a lot of things. But at the same time, we had to understand that maybe she (the mother) wasn't feeling alright…. I would say I think, us as midwives, if we did our jobs like during the night… [If] we didn't rest as in waiting for people to come and tell us their problems what they are facing, rather we go in doing ward rounds, you know… walk around and see how people are doing, maybe we could have seen that this mother was not feeding the baby the right way and we could have saved the baby." Tertiary hospital nurse-midwife<br><br>"The only problem I saw or as challenge, its like may be the mother who are not informed properly and they are feeding at night when they are sleeping and you find the baby has choked at night and you will just discover in the morning they are bringing you the baby who has died." Nursing officer at a district hospital
	Discussions of satisfaction with support	14	"What else can we say, so far we don't have any problems but maybe it would be better if it was three times sometimes, we know that they are busy but sometimes people are scared and want to ask something." Aunt from a district hospital

			"The reason why I am saying what they explained was not enough is that I have seen it that whenever some visitors come to congratulate us for the baby they tend to give her some lessons about breast feeding. After listening to them and when she started doing what she was told by those visitors I could see the difference in terms of how she could breast feed the child. This was good sign for me that at hospital she was not given enough education in regards to breast feeding." Father from the tertiary hospital<br><br>"I feel okay and I welcomed nicely because I did not know how I was going to breastfeed the baby but when the health practitioners told me about how to breastfeed a child I welcomed it and I used it." Mother from a district hospital
	Sources of support	61	"My roles are many. I have to make sure that I prepare food for the mother and I also help her with taking care of the baby like changing nappies, holding it. Then I also pick medicine for her from the nurse because she can't walk properly and I have to make sure that she drinks it also I make sure that at least she eats a fruit and vegetables because we were told that these are important for her first recovery as well as drinking plenty of water. So I make sure that all that is happening." Aunt from a district hospital<br><br>"They (nurses) told us that the babies should be exclusively breast fed… Since she (mother) is not well, they are explaining everything to me…" Grandmother from a district hospital<br><br>"So normally for these cases that we have noticed there are problems, we normally tell the guardians (mother's relative) to be on the alert and to be close by the patient." Clinical officer from a district hospital<br><br>"Some mothers prefer to have their relatives around with them, and also if the baby and the mother are both comfortable breast feeding becomes easier." Nurse officer from a district hospital

			"We have also what we call patients attendant who look after the babies and supervise the women when they are breast feeding the babies." Tertiary hospital nurse<br><br>"Sometimes they (mothers) share food, sometimes if someone is doing things the wrong way they do teach each other… You find out that some patients have some knowledge so they take advantage to teach others about breast feeding. " Clinical officer from a district hospital<br><br>"When am in postnatal ward… I check if the baby is being breastfed, if the milk production is normal and if the baby is being troubled upon breastfeeding, and the positioning of the baby when breastfeeding. " Clinical officer from a district hospital<br><br>"We normally monitor how this baby has been attached into the breast how this baby is breast feeding and if the lactation has been properly established by the mother, if the milk is coming out that's what we monitor, but we don't have that specific interval to say this is it, that will be a lie." Nurse from a district hospital
Other	Other	0	N/A
