# Supplementary material for: Beyond early initiation: A qualitative study on the challenges of hospital-based postpartum breastfeeding support
Source: PLOS Glob Public Health. 2022 Nov 8;2(11):e0001266. doi: 10.1371/journal.pgph.0001266 (PMC10021460; doi:10.1371/journal.pgph.0001266)
Supplement: S1 Text — (PDF) [file pgph.0001266.s003.pdf]

Healthcare workers experiences with interventions to improve neonatal health

Topic Guide

- *Introduce yourself and ask the participant how they are doing today, etc.*
- *Introduce the project and go through the consent form with the participant*
- *Get the participant to fill out the demographics form*

Topic	Question	If not already brought up by participant, probe:
**Introduction**	1. How long have you worked in the unit?	
	2. Which units do you prefer to work in?	
	3. Please describe a typical day in this ward for you	• What do you do?<br>• What are your responsibilities?
**Training**	4. First of all, can you tell me more about when you started providing breastfeeding support?	
	5. Can you tell me how you were trained?	• Formal or informal?<br>• Length of training?<br>• Who were the trainers?
	6. In your training, what did you find most helpful/useful for providing breastfeeding support in your workplace?	
	7. What other things would you like to learn to better be able to provide breastfeeding support in your workplace?	
	8. What stood out for you while learning about providing breastfeeding support such as anything you found surprising or interesting?	
**Initiation**	9. I have a couple general questions about providing breastfeeding support at your workplace. Would you describe to me how breastfeeding support is initiated at your workplace?	• Who is supposed to initiate?<br>• Who actually initiate it?<br>• Any differences between shifts (day/night/weekend/holidays)?
	10. Would you describe factors that make it easier to provide breastfeeding support? Can you provide an example?	
	11. Would you describe factors that make it more difficult to provide breastfeeding support? Can you provide an example?	
	12. What do you do when you face challenges? Can you provide an example?	• Sources of support?<br>• Shift differences?
	13. Would you describe any disagreements with i providing breastfeeding support that you may have observed or heard about?	• Who disagreed with who?<br>• Why did they disagree?<br>• What happened?<br>• How was it resolved?

		• Any other examples?
	14. Can you describe a time at your workplace when there was a delay in providing breastfeeding support after delivery?	• How long was the delay?<br>• Why do you think this happened?<br>• What resulted in your example?
	15. Can you describe a time at your workplace when breastfeeding support was not given?	• Why?<br>• What happened?
**Monitoring**	16. Would you describe how monitoring happens at your workplace?	• Who monitors?<br>• When and how often?<br>• What do they do to monitor?<br>• Where do they monitor?<br>• Ideal vs reality?<br>• Shift differences?
	17. Would you describe factors that make it easier to follow up on breastfeeding practices? Can you provide an example?	
	18. Would you describe factors that make it more difficult to follow up on breastfeeding practices? Can you provide an example?	
	19. What do you do when you face challenges? Can you provide an example?	• Sources of support?<br>• Shift differences?
	20. Would you describe any disagreements with monitoring breastfeeding that you may have observed or heard about?	• Who disagreed with who?<br>• Why disagree?<br>• What happened?<br>• How was it resolved?<br>• Any other examples?
	21. Can you describe a time at your workplace when there was a delay in monitoring?	• How long?<br>• Why?<br>• What happened?
	22. Can you describe a time at your workplace when monitoring did not happen?	• Why?<br>• What happened?
**Perceptions of health care professionals**	*23.* Would you describe how support was provided to postpartum mothers in your workplace before breastfeeding counselling?	
	24. What has changed since breastfeeding counselling was introduced?	
	25. In your experience, can you describe situations where providing breastfeeding support helped the baby get well?	• What happened?<br>• Why they think it happened?<br>• What could be learned from this situation?
	26. In your experience, can you describe situations where providing breastfeeding support caused harm to the baby?	• What happened?<br>• Why they think it happened?<br>• What could be learned from this situation?

		• Did it change your opinion on providing breastfeeding support?
	27. Overall, what do you think about providing breastfeeding support?	• How does using providing breastfeeding support make you feel?<br>• What did you like about using it and what did you not like?<br>• How has it influenced your practice?
**Perceptions of parents**	28. What are some of the perceptions that mothers/guardians have of breastfeeding support after delivery?	
	29. Why do you think they think this way?	
	30. How do you explain breastfeeding to the mother/guardian?	• Who talks to guardians?<br>• When/at what point<br>• Give an example of what you would say<br>• If no one talks to the parents then why not?
	31. Any examples of parents who refused? Who, why and what was done?	
	32. Any examples of parents who initially refused and then accepted? What changed their mind?	
	33. Any examples of parents who accepted right away? Why do you think this was?	
	34. Would you describe how parents care for their baby while breastfeeding after delivery?	• Holding, changing, feeding,<br>• Challenges<br>• How to support, peer support
	35. What factors do you think make it easier for parents to be comfortable with breastfeeding after delivery? Can you provide an example?	
	36. What factors do you think makes it more difficult for parents to breastfeed after delivery? Can you provide an example?	
**Closing**	37. Thank you. These are all the questions I had for you. Is there anything you would like us to know about your experience with providing breastfeeding support or how health care workers could be supported to continue to provide breastfeeding counselling?	
