# Supplementary material for: Beyond early initiation: A qualitative study on the challenges of hospital-based postpartum breastfeeding support
Source: PLOS Glob Public Health. 2022 Nov 8;2(11):e0001266. doi: 10.1371/journal.pgph.0001266 (PMC10021460; doi:10.1371/journal.pgph.0001266)
Supplement: S2 Text — (PDF) [file pgph.0001266.s004.pdf]

Caretaker experiences with interventions to improve neonatal health

Topic Guide

- *Introduce yourself and ask the participant how they are doing today, etc.*
- *Introduce the project and go through the consent form with the participant*
- *Get the participant to fill out the demographics form*

Topic	Question	If not already brought up by participant, probe:
**Introduction**	1. What is your relationship to the newborn?	
	2. Would you be able to tell us a little of yourself and where you are from?	• Where are you from?<br>• What is your/your husband's profession?<br>• How many children do you have?
	3. How did you come to the hospital today?	• What means of transport?<br>• How long did it take?<br>• Were you referred here by another facility? What was the process of referrals?
	4. Please describe if you experienced any challenges coming to the hospital today	• Who is looking after your other children at home?<br>• Cost of transport? Availability of transport?<br>• Concerns regarding time away from home and/or job?
	5. What would make it easier for you to come to the hospital today?	
**Initiation and monitoring**	6. Would you describe how medical staff initiated breastfeeding with you?	• How did that make the you (parent) feel?<br>• Were there any delays in initiating the intervention? If so, why did they think there was a delay?
	7. Would you describe how the medical staff explained breastfeeding to you?	• Was it sufficient? What did you want to know more?<br>• How did the information make you feel?
	8. Do you feel the staff were sufficiently trained to start and follow-up on breastfeeding?	• Why or why not?<br>• How did that make you feel?
	9. Would you describe how medical staff followed up on breastfeeding after delivery?	• How often did medical staff check in?<br>• Did you feel that was sufficient?
	10. What roles and responsibilities did parents have in looking after their baby while breastfeeding?	• How did medical staff support parents in their roles?

		• What made doing these activities difficult?
**Perceptions of parents**	11. When you first heard of breastfeeding, what did you think of it?	• What are your understanding of the intervention?<br>• What is said about the intervention among people in your family (i.e. husband, mothers-in-law, fathers-in-law… etc)?<br>• What is said about the intervention among people in your community and/or religious leaders?<br>• What are cultural beliefs around components of the intervention (i.e. breastmilk for lactation support, etc.)
	12. When you first heard breastfeeding, how did that make you feel?	• Any fears? Any feelings of hope? Joy? Etc.<br>• For especially lactation support: if HIV positive, did you have any specific concerns?
	13. Now that you have breastfed your baby, how do you feel about it now?	• Any changes in their opinions? What changes? How did it change? Why the change in opinion?
	14. What factors do you think make it easier for you to be comfortable with breastfeeding? Can you provide an example?	• What support can medical staff provide?
	15. What factors do you think makes it more difficult for you to breastfeed? Can you provide an example?	• What challenges do you face while using the intervention?
**Closing**	16. Thank you. These are all the questions I had for you. Is there anything you would like us to know about your experience with breastfeeding or how you could be supported to breastfeed your newborn?	
