# Supplementary material for: Beyond early initiation: A qualitative study on the challenges of hospital-based postpartum breastfeeding support
Source: PLOS Glob Public Health. 2022 Nov 8;2(11):e0001266. doi: 10.1371/journal.pgph.0001266 (PMC10021460; doi:10.1371/journal.pgph.0001266)
Supplement: Attachment — Submitted filename: Response to reviewers_R1.pdf [file pgph.0001266.s005.pdf]

THE UNIVERSITY OF BRITISH COLUMBIA

Faculty of Medicine
The University of British Columbia
317-2194 Health Sciences Mall
Vancouver, BC V6T 1Z3

Phone 604 822 2421
Fax 604 822 6061
www.med.ubc.ca

Dear editors and reviewers,

Re: "You just take it for granted that everything is alright": Beyond early initiation to challenges of continued facility-based breastfeeding support in Malawi (PGPH-D-22-00362)

On behalf of the authors, thank you for the positive and constructive feedback. We truly appreciate the time and effort of editors and reviewers to consider our manuscript as well as the thoughtful comments. We have gone through the comments in detail and revised the manuscript accordingly. A detailed point-by-point response to each comment is provided below:

Reviewer #1:

Overall this is a very good paper on an important topic of major global public health relevance. As such, it strongly deserves to be published as it can help inspire and inform:
a) Similar work in other settings
b) Follow-on research to address the many challenges identified here.

I have a few minor comments for authors to consider in order to further improve the value of their already strong, well described and well conducted study.

Title

1. Whilst I like the quote I wonder if dropping this and editing might help get more readers and more future citations. Title should ideally include: study type (qualitative); perhaps NOT mention study setting so as to broaden appeal (the messages in this paper for sure apply to many other settings, not just Malawi – see also https://www.natureindex.com/news-blog/studies-research-five-ways-increase-citation-counts

   Response: Thank you for the insightful comment and the reference. We have now updated the title to have a more broad appeal while being succinct, descriptive of the study type and use of a colon as recommended by article. The revised title is as follows, "Beyond early initiation: A qualitative study on the challenges of hospital-based postpartum breastfeeding support" (lines 1-3).

Abstract

2. Background might also be edited to make the point that the challenges faced in Malawi are in fact common to MANY other settings.

Response: Thank you for the recommendation to broaden the appeal of the study. We have added the following sentence to highlight that these concerns are common to many resource-limited health settings, “Improving breastfeeding practices is key to reducing child mortality globally. Sub-optimal exclusive breastfeeding rates may be associated with inadequate hospital-based postpartum breastfeeding support, particularly in resource-limited health settings such as Malawi.” (See lines 24-27).

3. Conclusions – might also mention clinical tools and approaches to help staff identify vulnerable infants who are at-risk of feeding/nutrition problems

   Response: Added to the conclusion, “We recommend the development of indicators to track continued facility-based breastfeeding, identify vulnerable infants at-risk of feeding problems, and strengthening care in postnatal wards, which is currently as neglected component of maternity care.” (See lines 47-48).

Background

Overall very good but could include some more references (see also last point re number of references cited). For instance:

4. Be explicit about the mortality/morbidity numbers from suboptimal BF e.g. “Increasing breastfeeding to near-universal levels for infants and young children could save over 800000 children’s lives a year worldwide, equivalent to 13% of all deaths in children under two, and prevent an extra 20000 deaths from breast cancer every year.” https://www.thelancet.com/series/breastfeeding https://www.evidentlycochrane.net/lancet-breastfeeding-series/

   Response: We have added the following sentence to be more explicit, “Following the WHO recommendations, increasing breastfeeding practices to near universal levels are estimated to prevent over 800,000 children’s deaths each year in low- and middle-income countries (LMICs), corresponding to 13.8% of deaths under two years old globally(2).” (See lines 61-63)

5. Discuss the role of BF towards global SDG targets http://waba.org.my/archive/breastfeeding-a-key-to-sustainable-development-unicef-world-breastfeeding-week-2016-message/

Response: We have added the following sentence to highlight the role of breastfeeding towards the Sustainable Development Goal targets, “As key to reducing child mortality, improving early health and nutrition, with life-long impacts on long-term health, cognitive development and participation in society, optimal breastfeeding practices play an important role in achieving Sustainable Development Goals to end poverty and fight global inequality (3–5).”(See lines 64-68).

In response to the recommendation for more references, three additional references were added to support this statement:

- Hansen K. Breastfeeding: a smart investment in people and in economies. The Lancet. 2016 Jan 30;387(10017):416.
- Lake A, Chan M. Breastfeeding: A Key to Sustainable Development [Internet]. 2016 [cited 2022 Jul 15]. Available from: http://waba.org.my/archive/breastfeeding-a-key-to-sustainable-development-unicef-world-breastfeeding-week-2016-message/
- Rollins NC, Bhandari N, Hajeebhoy N, Horton S, Lutter CK, Martines JC, et al. Why invest, and what it will take to improve breastfeeding practices? The Lancet. 2016 Jan 30;387(10017):491–504.

6. Give some definitions and a sentence or two extra re the differences between exclusive vs predominant vs partial BF: this is an important distinction and some readers might not be familiar with the issues. E.g. below is a good ref of the relative impact https://pubmed.ncbi.nlm.nih.gov/27013313/

   Response: Added to the introduction, “Exclusive breastfeeding is defined as the infant receiving only breastmilk, in contrast to partial breastfeeding where other liquids and/or solids are also provided (2). In comparison to exclusive breastfeeding, partial breastfeeding (RR 1.83, 95% 1.45-2.32) and no breastfeeding (RR 10.88, 95% CI: 8.27-14.31) is associated with significantly high risk of neonatal death (2).” (See lines 57-61).

   This refers to the article recommended by reviewer 1: Timing of initiation, patterns of breastfeeding, and infant survival: prospective analysis of pooled data from three randomised trials. Lancet Glob Health. 2016 Apr 1;4(4):e266–75.

7. Were there some original aims and specific objectives of this study – the “purpose” is expressed but is vague

Response: We have added the following to clarify, "…the objective of the secondary analysis was to better understand the availability and content of postpartum breastfeeding support with attention to early initiation of breastfeeding compared to post-initiation counselling" (See lines108-110).

Methods

8. Please say some more about the main study if this was really a secondary analysis. What were the aims/objectives of the wider 'parent' study and how do they differ from this one? Where are the results of the main study published?

   Response: Thank you for opportunity to clarify. We have added the following, including a reference to the primary study: "The objective of the primary study was to identify overall gaps to be addressed in order to improve breastfeeding support in Malawi (16), while the objective of the secondary analysis was to better understand the availability and content of postpartum breastfeeding support with attention to early initiation of breastfeeding compared to post-initiation counselling." (See lines 106-110)

   Publication of the primary analysis: Nyondo-Mipando, A.L., Kinshella, ML.W., Salimu, S. et al. Familiar but neglected: identification of gaps and recommendations to close them on exclusive breastfeeding support in health facilities in Malawi. Int Breastfeed J 16, 72 (2021). https://doi.org/10.1186/s13006-021-00418-9

Results:

9. A table summarising the key characteristics of the respondents would be helpful (e.g. years of experience for the HCW; age and education level of the mothers)

   Response: Thank you for the comment. Participant characteristics are described in more detail in the primary study (Nyondo-Mipando et al 2021, https://doi.org/10.1186/s13006-021-00418-9. See response to comment 8 for the full reference).

10. - On page 9, authors make an important point that though information is provided, mother's often forget/go back to old ways of doing things. Did they expand on what they thought were reasons for this? Was it for instance: the way info was provided (verbally/pictures/posters/what?) The quantity of info provided? Or just the fact that post-birth is such a busy and difficult time with so much going on? On a related note, did you have any information on whether antenatal

care/antenatal messages reduced this tendency to forget post-delivery counselling messages.

Response: Thank you for highlighting this important topic. Unfortunately, participants did not expand on reasons. However, we have dedicated a paragraph in the discussion to this issue where we propose that fatigue and low maternal education may be potential reasons why (see lines 374-389).

11. Please could authors also add a sentence and/or reference to say more about what the "counselling on best BF practices" actually involved. Was this part of general post-natal care guidance? If so, was this left up to each individual healthcare worker to decide what to do and say? Or are there locally tailored materials? Centre-specific guidelines? National Malawi Guidelines? International UNICEF or WHO or other guidelines?

    Response: We have reworked the paragraph to clarify "counselling mothers on breastfeeding practices" and that "Counselling messages varied between health workers but often included information on the benefits and frequency of breastfeeding. Some health workers also mentioned counselling mothers on correct positioning, attachment, and hygiene." Please see lines 215-219.

12. Page 10, Line 198. Can you say what an average time to discharge is after birth? IS there a minimum observation period before a mother/infant pair can be discharged? IS there any data on what the average stay in hospital post-delivery is in this setting?

    Response: We have reworked the paragraph to clarify the time spent with direct monitoring in the labour ward and added the overall average time to discharge: "Health workers highlighted a short two hour window of direct monitoring after delivery when the mother and infant were still in the labour ward before moving to the postnatal ward for recovery or another ward for further care in the case of complications. The average time to discharge after delivery for mothers and infants without complications was between 12-24 hours." Please see lines 233-236. To the best of our knowledge, we did not find published data reporting timing of hospital postpartum stay prior to discharge in Malawi.

13. Page 11, line 227 – can you say what training the HCW might have had (or what support materials they might have) to assess adequacy of breastfeeding. Were any tools or guidelines used for this (e.g. see this SR for a list of available BF assessment tools https://pubmed.ncbi.nlm.nih.gov/33628437/ - perhaps some of these might be recommended in the discussion of this paper)

Response: Thank you for raising this important point. Unfortunately, our study identified a lack of monitoring for breastfeeding in the postnatal ward, so HCW training to assess adequacy of breastfeeding or breastfeeding assessment tools were not discussed by participants. Once mothers were in the postnatal ward, the procedure was for mothers to seek out health workers if they had a problem in lieu of monitoring. We have reworked the paragraph to clarify this point, “While counselling and guidance to initiate may occur after delivery, there was a lack of routine monitoring and follow-up lacked observing breastfeeding practice.” See lines 240-242.

14. Page 13, “Feasibility, challenges and local solutions” – in the discussion to accompany this section (rather than in the results section here), it would be helpful to note any data on BF indicators from the hospitals studied here. Are these similar to or higher/lower than national average. This would help readers understand how typical these challenges are.

    Response: Thank you for raising this point. While it was not specific on breastfeeding, facility assessments to evaluate the quality of newborn care provided at study district hospitals were conducted with similar results to a larger assessment across Malawi, which suggests generalizability of these challenges. We have added the following line to the manuscript, “Facility assessments in our study district hospitals had similar results to a wider assessment using the same tool in five districts across Malawi suggesting generalizability of these challenges in neonatal and postnatal care (19).” Please see lines 417-419.

<u>Discussion</u>

15. Please add a few lines on possible short / long term policy and practice implications e.g. wider use of breastfeeding assessment tools such as noted above; more training for staff; dedicated BF support staff; beginning messaging about BF in the antenatal period rather than perinatally when there’s too much going on for mothers to take all on-board.

1. Response: Thank you for the opportunity to elaborate. We have added the following: “Exclusive breastfeeding could be increased through enhancing and investing in health facility capacity to support breastfeeding with continuity from antenatal care, counselling for early initiation, follow-up until hospital discharge and postpartum care visits (31). This would include revamping, expanding and institutionalizing the baby-friendly hospital initiative through more practical training of staff in breastfeeding protection, promotion and overall postpartum support (31,32). This training needs to emphasize continuity between the

antenatal and postnatal period, with attention to vulnerable populations like adolescents, primigravidas, those who delivered through caesarean section, family-centred care with involvement of husbands/partners and birth companions, and integration with programmes for the prevention of mother-to-child transmission of HIV (16,31,32). Staff capacity could be supported by task-shifting of the roles of education and supporting mothers with breastfeeding to lay health workers (16). There is evidence that a combination of professional support and peer support by trained and experienced lay health workers supports the continuation of breastfeeding, with the role of peer support identified to be the most important during the postnatal period (33,34). However, there is a gap in research on implementing breastfeeding peer support programs in LMICs, where health facilities may be resource-constrained and staffing capacity could be greatly bolstered by such programs (33,35). Additionally, strengthening staff capacity at the health facility can be accompanied by providing community-based strategies to support exclusive breastfeeding and implementing communication campaigns tailored to the local context (36). Malawi is resource-constrained and had halted recruitment of health care workers, which has prolonged the staff shortages. There are plans to revise the postpartum care under pipelines and this study will feed into those plans." See lines 423-443

References:

16. OK but a relatively small number. Consider this interesting paper https://www.nature.com/articles/news.2010.406 . It might both help future readers and citations of this paper to expand the number of references. What about, for example, mentioning some of the below:
- WHO Guidelines from here: https://www.who.int/health-topics/breastfeeding#tab=tab_1
- Other guidelines for providing support and managing problems for vulnerable infants e.g. https://www.ennonline.net/ife/iferesourcesoutputs
https://www.ennonline.net/mamicarepathway
https://www.cdc.gov/breastfeeding/resources/index.htm
https://www.globalbreastfeedingcollective.org/

Response: Thank you for the comment. We have increased the number of references to 36 including referencing the WHO as recommended.

Reviewer #2:

General comments: This is an interesting and informative article that sheds light on the "black-box" in health service delivery for breastfeeding support. The authors have done a

nice job of capturing not only health worker perspectives, but that of the new mothers and other family members.

Minor comments:

2. Line 54: not sure if (2015) refers to a reference or the year, please make clear.

   Response: Thank you for your attention to detail. We have reworked the sentence for clarity, “data from 2015 reported a shortfall between the early initiation rate (76%) and the rate of exclusive breastfeeding (61%) (8)”. Please see lines 76-77.

3. Reference 9 is missing journal information

   Response: This has been updated.

<u>Methods:</u>

4. Study design and Setting: Please provide more description of the facilities i.e. BFHI certification, size of the catchment area, size of nursing staff/midwives etc. It may also be useful to include a table with these characteristics.

   Response: Thank you for the recommendation. We have added a reference for further description of the facilities. Facility assessments assessing the quality of newborn care in the district hospitals was conducted as part of the overall IMCHA project. See lines 117-118 for the added sentence, “For facility assessments that further describe these district facilities and their quality of neonatal services provided, see Kawaza et al 2020 (19).”

5. Recruitment and selection: Although authors state purpose sampling, It seems as If there were two types of purposive sampling used one for the health workers and one for the mothers/family members. Can you please provide more clarity on the type of purposive sampling used i.e. convenience, criterion, used for each group?

   Response: Thank you for the opportunity to clarify. We have reworked the sentence for clarity to highlight that health workers engaged in decision-making and neonatal care, breastfeeding mothers and their relatives were purposively selected according to their roles (see line 129-130). The categories of health workers engaged in decision-making and neonatal care are further described in lines 132-134.

6. How was it determined that a sample size of 5-10 participants was sufficient for data saturation, was this from formative work? If so please indicate

   Response: We have added further information to clarify, "Based on the limited number of staff available for neonatal care, particularly at the district hospitals, a sample size of 5-10 health workers and 5-10 family members at each site were estimated to reach data saturation with a variety of perspectives." See lines 138-141.

7. Data collection: What was the rationale for including data from the pilot interviews? The questionnaires were adapted after piloting.

   Response: We included the data from the pilot interviews due to the exploratory nature of the study design. This has been added to the methods in line 153-154.

Results

8. Line 270-271 is repetitious of the quote in line 274. Could be more concise and allow the quote to make the point.

   Response: The line has been edited down for conciseness. See line 307-308.

9. Lines 323-327 is repetitious of the quote in line 328. Could be more concise and allow the quote to make the point.

   Response: The lines have been reduced for conciseness. See 360-363.

Discussion:

10. Please add to the discussion a paragraph explaining their results in the context of the Malawian health system and what are possible next steps e.g., are there are policies or program actions that government is undertaking to address staffing shortages? How about facility task shifting and/or community support for breastfeeding e.g., community health workers, peer networks etc.? Also breastfeeding support initiatives that include other family members.

    Response: Thank you for the opportunity to elaborate. We have added the following: "Exclusive breastfeeding could be increased through enhancing and investing in health facility capacity to support breastfeeding with continuity from antenatal care, counselling for early initiation, follow-up until hospital discharge and postpartum care visits (31). This would include revamping, expanding and

institutionalizing the baby-friendly hospital initiative through more practical training of staff in breastfeeding protection, promotion and overall postpartum support (31,32). This training needs to emphasize continuity between the antenatal and postnatal period, with attention to vulnerable populations like adolescents, primigravidas, those who delivered through caesarean section, family-centred care with involvement of husbands/partners and birth companions, and integration with programmes for the prevention of mother-to-child transmission of HIV (16,31,32). Staff capacity could be supported by task-shifting of the roles of education and supporting mothers with breastfeeding to lay health workers (16). There is evidence that a combination of professional support and peer support by trained and experienced lay health workers supports the continuation of breastfeeding, with the role of peer support identified to be the most important during the postnatal period (33,34). However, there is a gap in research on implementing breastfeeding peer support programs in LMICs, where health facilities may be resource-constrained and staffing capacity could be greatly bolstered by such programs (33,35). Additionally, strengthening staff capacity at the health facility can be accompanied by providing community-based strategies to support exclusive breastfeeding and implementing communication campaigns tailored to the local context (36). Malawi is resource-constrained and had halted recruitment of health care workers, which has prolonged the staff shortages. There are plans to revise the postpartum care under pipelines and this study will feed into those plans." See lines 423-443

11. Line 395-397: I think your argument here about generalizability to other facilities is weak as you've not adequately described these facilities.

    Response: Thank you for the opportunity to strengthen. We have added the following line to justify our statement, "Facility assessments in our study district hospitals had similar results to a wider assessment using the same tool in five districts across Malawi suggesting generalizability of these challenges in neonatal and postnatal care (19)." Please see lines 456-458.

Thank you. Please let us know if you need anything else.

Best regards,

M Kinshella

Mai-Lei Woo Kinshella, on behalf of the authors
